# Current Perspectives on Remifentanil-PCA for Labor Analgesia: A Narrative Review

**DOI:** 10.3390/medicina61091550

**Published:** 2025-08-29

**Authors:** Pia Vovk Racman, Miha Lučovnik, Tatjana Stopar Pintarič

**Affiliations:** 1 Clinical Department of Anaesthesiology and Intensive Therapy, UMC Ljubljana, 1000 Ljubljana, Slovenia; pia.vovk@kclj.si; 2 Department of Perinatology, Division of Obstetrics and Gynecology, UMC Ljubljana, 1000 Ljubljana, Slovenia; miha.lucovnik@kclj.si

**Keywords:** Remifentanil-PCA, labor analgesia, labor

## Abstract

Remifentanil is a potent opioid characterized by a unique pharmacokinetic profile that makes it well-suited for analgesia in obstetrics. When administered in a patient-controlled analgesia (PCA) modality, remifentanil has become a recognized and versatile alternative for labor pain relief in cases where epidural analgesia is contraindicated or is declined by the parturient. It offers mild to moderate pain relief, effectively decreasing pain from severe levels to a more manageable, moderate intensity. Remifentanil can be administered promptly and acts quickly, making it particularly useful in rapidly progressing or advanced labor. It can also benefit women with anxiety or tokophobia, as its sedative, anxiolytic, and euphoric effects help reduce pain perception and facilitate coping during labor. While it is not superior to epidural analgesia in terms of analgesic efficacy, remifentanil-PCA has obtained a role as a complementary pain-relieving option in several obstetric situations. Remifentanil-PCA is associated with high patient satisfaction, which is closely linked to realistic counseling and proper expectation management. The safety profile for both mother and neonate has been established; however, safety depends on cautious incremental dosing tailored to sedation levels, the use of supplemental oxygen, rigorous monitoring, and avoiding background infusion. Vigilant supervision by healthcare providers is essential, ideally supported by the continuous presence of an anesthesia team in the labor ward.

## 1. Introduction

Labor pain is considered to be one of the most intense forms of pain a woman may experience; however, it is a natural and expected part of childbirth, rather than a pathological condition [1]. Effective management of this pain is a vital component of modern perinatal care. In this context, epidural analgesia (EA) is widely regarded as the gold standard for pain relief during labor due to its proven efficacy and safety [2]. Besides EA, various other options are available. Pharmacological alternatives include systemic opioids and nitrous oxide, while nonpharmacological methods encompass relaxation techniques, manual therapies, acupuncture, and transcutaneous electrical nerve stimulation [3].

Remifentanil is an ultra-short-acting synthetic opioid that acts as an agonist of μ-opioid receptors. It has a rapid onset of action, approximately one minute, and a short context-sensitive half-time of three minutes, owing to its fast degradation by non-specific tissue and plasma esterases. Although it crosses the placenta, it undergoes rapid metabolism in the fetus, which may make it safer for the fetus compared to other opioids. With these promising features, remifentanil emerged as an option for labor analgesia in the early 2000s [4,5,6]. Although it is still used off-label, it has become an established choice for labor analgesia over the past two decades [7,8]. It is administered intravenously via a patient-controlled analgesia (PCA) modality, which can replicate the intermittent profile of labor contractions [9].

The aim of this paper is to provide a review of analgesic efficacy of remifentanil-PCA for labor analgesia, discuss parturient satisfaction and the safety of remifentanil PCA, and report our extensive experience with the use of remifentanil-PCA for labor analgesia at the Department of Perinatology, Gynaecology clinic, UMC Ljubljana [9,10,11,12,13,14].

## 2. Methods

### 2.1. Search Strategy

US National Library of Medicine, National Institutes of Health (www.pubmed.gov) was searched using the keywords “remifentanil” and “labor”. No limitations were placed on publication date or article type. The last search was performed on 28 July 2025.

### 2.2. Inclusion and Exclusion Criteria

Randomized controlled trials and meta-analyses with the presence of detailed clinical data that were available in full text articles were included in this narrative review. Abstracts of unpublished studies, case reports, letters, editorials and articles missing full text were excluded.

### 2.3. Analgesic Efficacy of Remifentanil-PCA Compared to Other Labor Pain Relief Options

Substantiated by multiple studies, remifentanil-PCA provides mild to moderate pain relief with a reduction in the Visual Analog Scale (VAS) pain score, ranging from −1 point in the study of Volmanen et al. to −5 points in the study of Evron et al. [14,15,16,17]. The mean reduction in VAS pain score is −2.8 points according to meta-analysis by Stourac et al., meaning it reduces pain from severe, unbearable (VAS 8–10) to intermediate, tolerable (VAS 5–7), which helps women to cope better with labor pain [9,14,15]. Reported dosage regimen in the literature usually ranges from 10 to 50 µg boluses with a lockout period of 2 min with or without background infusions [7,9,10,11,12,13,14,18].

Analgesic efficacy of remifentanil-PCA is inferior to that of EA, confirmed by three meta-analyses [19,20,21], and superior to nitrous oxide [22] and other systemic opioids [23,24,25,26,27]. According to meta-analysis by Liu et al., the effect size difference between EA and remifentanil-PCA is three points on VAS [21]. However, compared to EA, remifentanil-PCA possesses qualities that may be preferable in some situations. Firstly, it offers an acceptable pain relief when neuraxial analgesia is contraindicated or not preferred by the parturient [10,15,16,17,20,28,29,30]. Secondly, its simple application, rapid onset of action, and non-invasive nature enable a faster onset of pain relief, making it particularly suitable for rapidly progressing or advanced labor (multiparas) [31,32].

In the RESPITE trial, which compared pethidine with remifentanil-PCA for labor analgesia, the proportion of women requiring conversion to epidural analgesia (EA) was significantly lower with remifentanil-PCA than with pethidine (19% vs. 41%). Additionally, women receiving remifentanil-PCA reported lower mean VAS pain scores compared to pethidine (50 vs. 65), while the reduction in VAS scores was similar in both groups (−25% reduction) [18]. Women in the remifentanil group were more satisfied with their pain relief when compared to those in the meperidine group, while no differences were observed in the overall birth satisfaction between the groups. This substantiated the superior analgesic efficacy of remifentanil-PCA from earlier studies comparing remifentanil-PCA and pethidine [17,23,25,27].

Nitrous oxide shares several characteristics with remifentanil-PCA for labor analgesia, including its ability to modulate the parturient’s perception of pain, its anxiolytic effects, the self-administration feature that provides a sense of control, and its rapid onset of action [33]. Volmanen et al. conducted a study comparing remifentanil-PCA to nitrous oxide. Despite a low sample size, the study demonstrated that remifentanil-PCA offers superior analgesia, as reflected in pain relief scores of 2.5 for remifentanil and 0.5 for nitrous oxide [22].

Notably, remifentanil-PCA does not provide complete pain relief but rather modifies pain perception by attenuating nociceptive transmission through μ-opioid receptor mechanisms. As such, it is not suitable for women seeking an entirely pain-free labor. Instead, it is more appropriate for individuals who prefer to avoid neuraxial analgesia or who wish to maintain some control over their labor, accepting moderate pain relief rather than total pain elimination.

Overall, the distinctive features of remifentanil-PCA make it a valuable option for labor pain management, especially in situations where anxiety or fear is prominent. The ability to modulate pain perception—despite potentially higher pain scores—highlights its role as a flexible, context-sensitive, and complementary tool alongside traditional analgesic methods such as epidural anesthesia [16,30,34,35].

Table 1 presents randomized controlled trials that evaluated the efficacy of remifentanil-PCA compared to other methods for labor analgesia. Table 2 presents four meta-analyses that studied the analgesic efficacy of remifentanil-PCA.

### 2.4. Parturient Satisfaction

The relationship between childbirth satisfaction, labor pain, and analgesia is complex [43]. Beyond the perception of labor pain itself—which is highly individual and intrinsic to each parturient at a given time—pain intensity during labor is influenced by various factors such as parity, labor duration, maternal pelvic anatomy, fetal size, fetal presentation, and labor augmentation [9,44]. A meta-analysis indicated that women who received remifentanil-PCA reported levels of satisfaction similar to those who received epidural analgesia (EA), despite having higher VAS scores at one hour compared to the EA group [20]. In a study by Dickinson et al., intrapartum satisfaction with pain relief was significantly higher with EA than with non-epidural techniques; however, overall birth satisfaction remained high regardless of the analgesic method used—highlighting that factors beyond pain relief also significantly influence the childbirth experience [9,45]. Furthermore, pain intensity and relief do not necessarily play principal roles in overall satisfaction unless expectations for either are unmet [43]. This highlights the importance of providing thorough and accurate counseling to parturients about the characteristics, benefits, and limitations of remifentanil-PCA, to align expectations with its effects—particularly emphasizing that complete pain relief is unlikely [9]. Additionally, involvement in decision-making and the timely administration of analgesia upon request are important factors contributing to overall satisfaction, both of which can be effectively facilitated with remifentanil-PCA [43]. One-to-one care, which is necessary for the safe administration of remifentanil-PCA, further enhances satisfaction [9]. Compared to pethidine, women receiving remifentanil-PCA reported greater satisfaction with pain relief; however, there was no significant difference in overall birth satisfaction [18]. Moreover, Logtenberg et al. observed a trend toward higher satisfaction among multiparous women using remifentanil-PCA compared to epidural analgesia (EA), suggesting that remifentanil may be a preferred option for this subgroup—particularly in short or rapidly progressing labors [30].

### 2.5. Maternal Side Effects and Potential Complications

Excessive sedation and consequent respiratory depression are well known side effects of parenteral opioids including remifentanil. Although parturients using remifentanil-PCA desaturate regularly, most oxygen desaturation episodes are of a short duration and self-limiting [10,46]. Of note are changes in breathing patterns that are common during labor, as stress and pain may cause hyperventilation. Contrarily, hypoventilation and desaturation occur after the contraction, due to a reduction in functional residual capacity combined with increased oxygen demands from the fetus and uterine contractions [46,47,48]. All of this has also been observed in women with epidural analgesia or no analgesia, but it was more profound with opioids and/or nitrous oxide [46,47]. Even though supplementary oxygen reduces the frequency of desaturation episodes; it does not affect their severity or duration [46] and may not prevent hypoxia [7]. This emphasizes the need for standardized protocols, along with supplementary oxygen administration.

More apnea events and a higher incidence of transient oxygen desaturation are reported with remifentanil compared to neuraxial analgesia [7,30,41]. When compared to nitrous oxide, remifentanil-PCA had higher subjective sedation scores and slightly more desaturations, which were not clinically relevant [22]. Reported rates of respiratory depression in remifentanil compared to pethidine vary between studies. Douma et al. found higher rates of sedation and oxygen desaturation in the remifentanil-PCA group [23], while the results of the RESPITE trial showed no difference in sedation or respiratory depression between groups [18]. Even though the dose of remifentanil was the same in both studies (40 µg), the dose of comparator pethidine was lower in the study of Douma et al., which could possibly explain higher rates of sedation and desaturation in the remifentanil-PCA group [18,23]. However, there was a greater incidence of low oxygen saturation when breathing room air and a higher need for supplemental oxygen in the remifentanil-PCA group in the RESPITE trial; the latter most likely represents caution on the part of clinical staff. Furthermore, low maternal oxygen saturation did not result in adverse maternal or neonatal sequelae [18].

Case reports of serious maternal adverse effects of remifentanil-PCA, such as cardiorespiratory arrest, can be found in the literature. However, closer examination revealed that these incidents were not caused by remifentanil-PCA itself, but rather by factors such as insufficient experience with the method and mismanagement, including accidental administration of ten times the intended concentration [49], prior use of long-acting opioids [50], and inadequate sedation monitoring.

Mild maternal adverse effects, such as nausea and pruritus, often require little to no intervention. Compared to neuraxial analgesia, no differences were found in the rate of nausea or pruritus using remifentanil-PCA [7]. Furthermore, when compared to pethidine, the need for antiemetic administration was significantly lower in the remifentanil-PCA group [18].

### 2.6. Safety Concerns

In addition to adherence to standard protocols, frequent clinical use of remifentanil-PCA is essential for ensuring safety, as regular practice helps integrate it into routine care and reduces the likelihood of errors [51]. Prompt management of side effects and prevention of serious adverse events—such as oversedation leading to respiratory arrest, severe desaturation requiring bag-mask ventilation or intubation, or cardiorespiratory arrest—should be achieved through standardized maternal monitoring. This monitoring should include pulse oximetry, heart rate, respiratory rate, capnography, and regular assessment of sedation levels, with the goal of ensuring that the parturient remains awake (Ramsay sedation score ≤ 2 on a 0–5 scale). Additional safety measures include continuous midwifery supervision and the presence of an anesthesiology team in the labor ward, enabling real-time dosage adjustments based on the parturient’s needs and sedation level, as well as prompt intervention in cases of side effects or complications [10,52].

**Figure 1:**
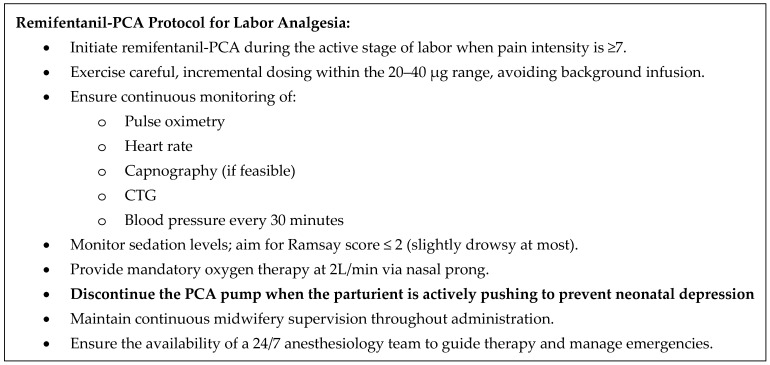
Suggestion of minimum institutional requirements for safe use of remifentanil-PCA.

Remifentanil-PCA has a limited analgesic efficacy. Studies have frequently shown that simply increasing the dose does not directly correlate with better pain control. Excessive increase in the dose of remifentanil frequently results in oversedation rather than lower pain scores, emphasizing the importance of careful dosage management and individualized care [18,29,53]. Finally, the degree of complications, notably oversedation and desaturation with respiratory depression, is expertise and resource dependent. The former improves with frequency of use. Regarding the latter, even in limited-resource environments, it would be beneficial to define minimum institutional requirements that must be met to ensure safe use of remifentanil-PCA (Figure 1).

Even though the risk for neonate appears to be minimal, the potential for remifentanil-PCA-associated neonatal adverse effects remains. Therefore, safety measures should include discontinuing remifentanil-PCA during active pushing, ensuring the availability of oxygen and the opioid antagonist naloxone, and having a pediatrician present [7]. As part of our institutional safety measures, we try to avoid the use remifentanil-PCA in labor earlier than 35 weeks of gestation because of unknown potential for adverse effects in preterm neonates.

### 2.7. Association with the Progress of Labor and Neonatal Outcomes

Remifentanil-PCA showed some advantages over neuraxial analgesia in labor and delivery outcomes [11]. In a cohort study of over 10,000 deliveries at our institution (Department of Perinatology, Gynaecology clinic, UMC Ljubljana, Slovenia) comparing epidural analgesia to remifentanil-PCA over five years, remifentanil-PCA was associated with lower rates of cesarean sections and operative vaginal deliveries in nulliparous women with both spontaneous and induced labor, as well as in multiparous women with spontaneous onset of labor. Moreover, remifentanil-PCA was associated with a lower incidence of operative delivery with pathologic CTG (cardiotocography) in all four studied groups. However, no differences in APGAR < 7 at 5 min, neonatal asphyxia, and neonatal intensive care unit admission were recorded between the two analgesic techniques within any of the studied groups. Nevertheless, the associations observed in that study may not necessarily imply a causal relationship. Favorable results of non-operative delivery with remifentanil-PCA may also point to the fact that more complicated labors require EA to assist in their management. Indeed, nulliparous women with EA were older, which represents an independent risk factor for labor dystocia. The reason could be that women with normal labor progress or expectations of faster labor are more likely to choose remifentanil-PCA to avoid the potential adverse/side effects of EA. This is particularly true of multiparous women, who can combine a fast delivery with rapid availability and a short use of pain relief [30]. Lower operative vaginal delivery rates were also observed in the RESPITE study, comparing remifentanil-PCA with intramuscular pethidine. However, this difference may not be directly attributable to remifentanil, but rather to its lower rate of conversion to EA, as EA itself is associated with a higher operative vaginal delivery rate [18].

As they cross the placenta, intravenous opioids carry a risk of neonatal sedation and asphyxia. In this context, remifentanil-PCA has shown promising results. A meta-analysis found no difference in Apgar scores at 1 and 5 min between remifentanil-PCA and epidural analgesia (EA) [21]. Additionally, a recent retrospective cohort study observed that signs of fetal asphyxia were less common with remifentanil-PCA compared to EA [54]. When compared to pethidine, no significant differences were noted in neonatal Apgar scores at 1 and 5 min, nor in the number of neonates with fetal acidosis or those transferred to higher levels of care [18,24]. However, the existing literature on the pharmacokinetics of remifentanil in neonates—particularly preterm infants—is limited and largely based on unverified assumptions derived from general review concepts [55]. A recent meta-analysis [54] concluded that neonates exposed to remifentanil in utero exhibited good adaptation to extrauterine life, with no evidence of perinatal asphyxia. The analysis included ten studies of fetal exposure to remifentanil, four of which evaluated labor analgesia and the remaining six cesarean sections. None of the neonates in these studies experienced hypoxemia, and there is no evidence linking remifentanil exposure to an increased risk of Apgar scores below seven at 5 min. When neonatal respiratory depression occurs, it is typically resolved within minutes, without the need for prolonged resuscitation. Remifentanil use for procedural analgesia in preterm neonates (>25 weeks gestation) appears safe, although its safety profile is limited by the potential for chest wall rigidity. Nonetheless, there is virtually no evidence regarding the safety or efficacy of remifentanil-PCA for analgesia in preterm labor.

### 2.8. Obstetric Considerations

Certain obstetric conditions, such as a history of previous cesarean delivery (CD), twin gestation, or a breech presentation, may pose heightened risks with epidural analgesia, prompting a preference for alternative analgesic approaches [56,57,58,59,60,61]. In our institution, a retrospective analysis of 127 planned vaginal breech and 244 twin deliveries for the period 2013–2021 was performed using data obtained from the Slovenian National Perinatal Information System. No statistically significant nor clinically relevant differences between the EA and remifentanil-PCA groups were observed in the rates of CD in labor, postpartum hemorrhage, obstetric anal sphincter injury, an Apgar score of <7 at 5 min after birth, birth asphyxia, and neonatal intensive care admission. This suggests that both EA and remifentanil-PCA are safe and comparable in terms of labor outcomes in singleton breech and twin deliveries [12].

In parturients with remifentanil-PCA who require CD, the selection of anesthetic technique for emergency CD is frequently unpredictable due to various factors, including individual patient preferences, obstetric considerations, potential contraindications to specific techniques, and the presence of labor pain, which can complicate the performance of neuraxial anesthesia. In the study evaluating the relationship between labor analgesia modalities and types of anesthetic techniques in categories two and three intrapartum cesarean section, remifentanil-PCA was associated with a higher incidence of general anesthesia in categories 2 and 3 emergency CD. The observed association might have been potentially influenced by the fact that women opting for remifentanil-PCA were often presented with either contraindications or refusal for EA [13].

### 2.9. Off-Label Use and Informed Consent

The use of remifentanil-PCA for labor analgesia remains off-label, making informed consent mandatory prior to its application. Parturients should be informed that remifentanil-PCA is unlicensed for labor analgesia; this does not mean it is unsafe, but rather that its use for this purpose falls outside the scope of its approved licensing. The consent process should include a clear explanation of the mode of administration (self-controlled, patient-administered medication), the limited analgesic effect with a low likelihood of complete pain elimination (to align expectations and increase satisfaction), as well as potential risks and side effects—such as respiratory depression, sedation, nausea, dizziness, and rare but serious complications like respiratory compromise. Additionally, anesthesia team monitoring and safety measures must be emphasized.

Given the increased likelihood of requiring general anesthesia in women who receive remifentanil-PCA, it is essential to counsel parturients beforehand about the possibility of transitioning to general anesthesia and the associated risks, particularly the higher risk of gastric paresis leading to regurgitation and aspiration if an urgent cesarean delivery becomes necessary [13].

### 2.10. Limitations of Reviewed Evidence

As demonstrated in Table 1, many studies are limited by small sample sizes. Additionally, there is considerable heterogeneity in remifentanil and comparator dosing protocols across studies. A notable gap in the research is the lack of detailed information on monitoring procedures used to prevent adverse events for both mother and neonate. Furthermore, data on serious adverse outcomes—such as respiratory depression, cardiocirculatory events, and mortality—are scarce, largely due to their rarity. These factors affect the generalizability of the findings, and potential publication bias may further influence the results.

Future multicenter research should primarily focus on best practices for maternal and neonatal monitoring, including the feasibility, safety, and cost-effectiveness of remote or centralized monitoring systems, which will increase the safety while reducing the need for constant parturient surveillance.

### 2.11. Our Experience

**Figure 2:**
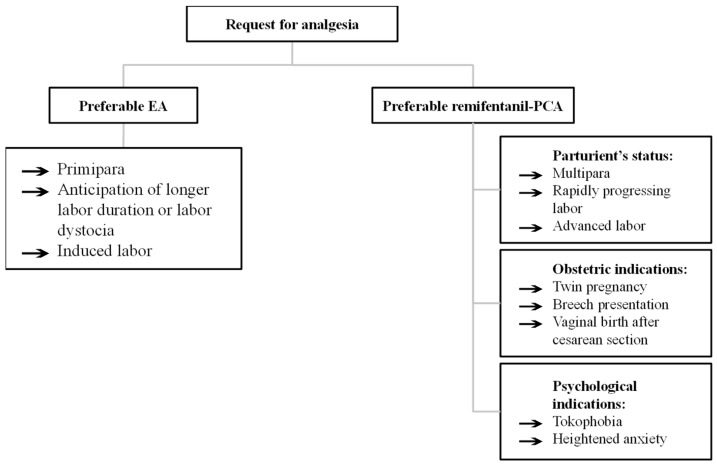
Decision-making suggestions for labor analgesia options according to institutional practice.

Evidence suggests that remifentanil-PCA provides the most effective non-neuraxial labor analgesia with good parturient satisfaction and adequate safety profile. At our institution (Department of Perinatology, Gynaecology clinic, UMC Ljubljana, Slovenia), remifentanil-PCA has been implemented in practice for labor analgesia in 2008 and used routinely since 2013, which amounts to 17 years of experience. By 2025, we had performed over 12,000 remifentanil applications. The entire extended obstetric team, including midwives, obstetricians, anesthesiologists, and nurses, possesses the expertise in the use of remifentanil-PCA for labor analgesia. Indications for remifentanil-PCA are parturient requests, cases where EA is contraindicated, unsuccessful epidural administration, accidental dural puncture, technical failure, advanced or rapidly progressing labor, and obstetric indications such as breech presentation, twin deliveries, or trial of labor after cesarean section (Figure 2). Contraindications include parturient refusal, a history of opioid allergy, and prior administration of parenteral opioids within the previous four hours. Given the “off-label” nature of remifentanil-PCA use as labor analgesia, informed consent is obtained prior to administration of medication. We have a standard operating protocol in place: dose range of boluses is 20–40 µg with a 2 min lock-out period and no continuous background infusion. We start at the lower end of the dose range (adjusted higher for multiparas) and continue with a stepwise approach as the labor progresses. The bolus is increased if pain intensity increases and a parturient respiratory rate is >9 breaths/min, oxygen saturation (SpO_2_) ≥ 94%, heart rate > 50/min and sedation score (scale 1–5: 1 = alert, 2 = slightly drowsy, 3 = drowsy, 4 = very drowsy, 5 = unarousable) [9]. As a safety measure, we routinely administer supplemental oxygen (2 L/min) via nasal catheter and continuously monitor the level of sedation (≤2 on a five-point categorical scale), oxygen saturation, heart rate, end-tidal carbon dioxide and CTG. We measure blood pressure every 30 min. To decrease the neonatal side effects, remifentanil-PCA is discontinued when the parturient is actively pushing to deliver the baby, or in case of a pathologic CTG. We strive to provide one-to-one midwifery care, and we always have an anesthesiology team available in the labor and delivery ward [10].

## 3. Conclusions

Over the past 20 years, remifentanil-PCA has established itself as a valuable and versatile alternative for labor pain relief worldwide, with usage rates and clinical experiences varying across regions. It is particularly favored when epidural anesthesia (EA) is contraindicated or declined by the parturient. Its rapid onset and capacity to deliver moderate, consistent pain relief quickly make it especially suitable for rapidly progressing or advanced labor. Additionally, remifentanil-PCA shows promise in obstetric situations associated with increased risks for intrapartum cesarean sections when EA is either unsuitable or avoided, such as breech presentations, twin deliveries, or trials of labor after cesarean. Its sedative, anxiolytic, and euphoric effects can also help reduce pain perception, providing benefits for women with tokophobia or heightened anxiety. Overall, remifentanil-PCA functions not merely as an alternative but as an important complementary tool within the limited spectrum of labor analgesia options. High satisfaction rates are often reported when clinical outcomes meet patient expectations, underscoring the importance of thorough counseling about its characteristics, advantages, and potential limitations. The safety profile of remifentanil-PCA heavily depends on careful incremental dosing without continuous infusion, rigorous monitoring—including regular assessment of sedation levels—and vigilant supervision by healthcare providers. Ideally, this is supported by the constant presence of an anesthesia team in the labor ward, prepared to promptly manage any side effects or complications.

## Figures and Tables

**Table 1:** Remifentanil RCTs that evaluated its efficacy for labor analgesia.

Study	Remifentanil-PCA Dose	Comparator	Result—Analgesic Efficacy
Douma et al., 2010, N = 180 [23]	LD: 40 µg + B 40 µg LO: 2 min	Pethidine IV LD 49.5 mg + B 5 mg LO 10 min Fentanyl IV LD 50 µg + B 20 µg LO 5 min	Remifentanil provided superior analgesia to pethidine and fentanyl during the 1st hour
Evron et al., 2005, N = 88 [17]	B: 20–70 µg LO: 3 min	Pethidine IV 75 mg (+ 75 mg + 50 mg if needed)	Remifentanil resulted in significantly lower VAS scores
Ng et al., 2011, N = 69 [25]	B: 25–30 µg LO: 3.75–4.5 min	Pethidine IM 50–75 mg	Remifentanil provided significantly lower pain scores
Blair et al., 2005, N = 39 [26]	B: 40 µg LO: 2 min	Pethidine IV B 15 mg LO 10 min	No difference
Volikas et al., 2001, N = 17 [27]	B: 0.5 µg/kg LO: 2 min	Pethidine IV 10 mg LO 5 min	Remifentanil resulted in significantly reduced hourly VAS
Stourac et al., 2014, N = 24 [28]	B: 20 µg LO: 3 min	EA: bupivacaine 0.125% + sufentanil 0.5 µg/mL LD 10 mL + 5 mL/60–90 min	No difference
Tveit et al., 2012, N = 37 [36]	B: 0.15 µg/kg + increase 0.15 µg/kg until relief LO: 2 min	EA: ropivacaine 0.1% + fentanyl 2 µg/mL LD 10 mL + inf 5–15 mL/h + rescue 5 ml	Remifentanil was inferior to EA
Volmanen et al., 2008, N = 45 [16]	B: 0.1–0.9 µg/kg LO: 1 min	EA: levobupivacaine 0.625 mg/mL + fentanyl 20 µg/mL B 20 ml	Remifentanil was inferior to EA
Douma et al., 2011, N = 20 [37]	B: 40 µg LO: 2 min	0.2% ropivacaine B 12.5 mL + 0.1% ropivacaine + sufentanil 0.5 µg/mL inf 10 mL/h	Remifentanil was inferior to EA
Ismail et al., 2012, N = 1140 [38]	B: 0.1/0.9 µg/kg LO: 1 min	EA: 0.125% levobupivacaine + 2 µg/mL fentanyl 8 mL LD + inf 8 mL/h CSE: B spinal levobupivacaine 2 mg + fentanyl 15 µg; epidural 0.125% levobupivacaine + 2 µg/mL fentanyl 8 mL LD + inf 8 mL/h	CSE was superior to EA and remifentanil
Stocki et al., 2014, N = 39 [29]	B: 20–60 µg LO: 1–2 min	EA: 0.1% bupivacaine + fentanyl 2 µg/mL LD 15 mL + inf 5 mL/h + rescue 10 mL/20 min	Remifentanil was inferior to EA
Balcioglu et al., 2007, N = 60, [39]	Compared two background infusions; Fixed B: 0.15 µg I_1_: 0.1 µg/kg/min I_2_: 0.15 µg/kg/min	Not applicable; compared two background infusions of remifentanil	Higher background infusion provided lower VAS scores
Balki et al., 2007, N = 20, [40]	Compared two dosing regimens; B_1_: 0.25 µg/kg + I_1_: 0.025–0.1 µg/kg/min B_2_: 0.25–1 µg/kg + I_2_: 0.025 µg/kg/min	Not applicable; compared two dosing regimens of remifentanil	No significant difference between groups in pain scores
Freeman et al., 2015, N = 1414 [41]	B: 20–40 µg LO: 3 min	According to local protocol	Remifentanil resulted in significantly higher mean pain intensity scores
Logtenberg et al., 2016, N = 409 [30]	B: 30 µg LO: 3 min	0.2% ropivacaine 12.5 mL LD + inf 0.1% ropivacaine + sufentanil 0.5 µg/mL	Remifentanil resulted in significantly higher pain intensity scores
Volmanen et al., 2005, N = 15 [22]	B: 0.4 µg/kg LO: 1 min	50% N_2_O	Remifentanil provided significantly better pain-relieving scores
Thurlow et al., 2002, N = 36 [42]	B: 20 µg LO: 3 min	Pethidine IM 100 mg	Remifentanil provided significantly better pain-relieving scores
Wilson et al., 2018, N = 400 [18]	B: 40 µg LO: 2 min	Pethidine IM 100 mg/4 h max 400 mg	Remifentanil resulted in a significantly greater reduction in median VAS scores

Abbreviations: N = sample size; B = bolus; I = background infusion; PCA = patient-controlled analgesia; LD = loading dose; LO = lock out; IV = intravenous; IM = intramuscular; EA = epidural analgesia; CSE = combined spinal epidural analgesia; inf = continuous infusion; VAS = visual analog scale; RCT = randomized controlled trial; N_2_O = nitrous oxide.

**Table 2:** Meta-analyses of analgesic efficacy of remifentanil-PCA.

Meta-Analysis	N of RCTs	Analgesia Methods	Conclusions
Lee et al., 2017 [20]	8	Remifentanil-PCA vs. EA	Significantly higher average VAS scores and VAS at 1 h in remifentanil-PCA Effect size difference: 1.33 points (95%CI 0.3 to 2.36)
Liu et al., 2014 [21]	5	Remifentanil-PCA vs. EA	Remifentanil inferior to EA for analgesic efficacy measured by VAS scores Effect size difference: 3 points (95%CI 0.7 to 5.2)
Schnabel et al., 2012 [19]	12	Remifentanil-PCA vs. EA, pethidine, N_2_O, fentanyl	Remifentanil-PCA superior to pethidine, inferior to EA for analgesic efficacy
Stourac et al., 2016 [15]	15	Remifentanil-PCA	Significant decrease in VAS (−2.826 points) during the first hour

Abbreviations: PCA = patient-controlled analgesia; EA = epidural analgesia; VAS = visual analog scale; RCT = randomized controlled trial; N_2_O = nitrous oxide.
